# Democratizing Glaucoma Care: A Framework for AI-Driven Progression Prediction Across Diverse Healthcare Settings

**DOI:** 10.1155/joph/9803788

**Published:** 2025-03-11

**Authors:** Cansu Yuksel Elgin

**Affiliations:** Department of Ophthalmology, Istanbul University-Cerrahpasa, İstanbul, Turkey

**Keywords:** artificial intelligence, disease progression, glaucoma, machine learning, personalized medicine

## Abstract

**Purpose:** To propose a conceptual framework for an AI-driven personalized glaucoma progression prediction system that integrates diverse data sources to enhance clinical decision-making and improve patient outcomes. This framework is specifically designed to address healthcare disparities in glaucoma care through scalable AI technology that can function across diverse resource settings, from tertiary care centers to remote clinics. The system aims to democratize access to expert-level glaucoma care while addressing challenges of bias, equity, and accessibility.

**Methods:** The paper outlines a comprehensive framework consisting of four main components: (1) data integration and preprocessing, (2) AI model architecture and training, (3) personalized prediction generation, and (4) a clinical decision support interface. The framework leverages multimodal neural networks to analyze structural imaging data, functional test results, clinical measurements, and patient demographics.

**Results:** The proposed framework addresses current limitations in glaucoma progression prediction by capturing complex interactions between various risk factors. Potential benefits include early detection of rapid progressors, optimized treatment strategies, improved patient counseling, and support for clinical research. Implementation challenges such as data quality, model interpretability, workflow integration, regulatory approval, and ethical considerations are discussed along with strategies to address them.

**Conclusions:** The AI-driven framework for glaucoma progression prediction represents a significant advancement in personalized glaucoma management. While challenges remain, the potential benefits in terms of preserved vision, improved quality of life, and more efficient healthcare delivery are substantial. Future research directions include incorporating genetic data, advanced imaging modalities, and federated learning techniques to further enhance the system's capabilities and impact.

## 1. Introduction

Glaucoma, a group of progressive optic neuropathies, remains a leading cause of irreversible blindness worldwide. It affects over 76 million people globally and is projected to impact 111.8 million by 2040 [1]. The disease is characterized by the progressive loss of retinal ganglion cells and their axons, leading to structural changes in the optic nerve head and functional decline in the visual field (VF) [2].

The global burden of glaucoma disproportionately affects underserved populations. Studies have shown significant disparities in glaucoma care access, diagnosis, and outcomes across different demographic groups. For instance, African Americans are six to eight times more likely to develop glaucoma than Caucasians and tend to develop it at a younger age [3]. Similar disparities exist across socioeconomic strata and geographic regions. A study by the National Eye Institute showed that individuals in rural areas and those from lower socioeconomic backgrounds experience longer delays in diagnosis and have poorer outcomes [4]. These disparities are often exacerbated by limited access to specialized equipment and expertise, highlighting the urgent need for more accessible and equitable approaches to glaucoma care.

The ability to predict glaucoma progression accurately is crucial for several reasons. First, it allows for timely intervention in rapidly progressing cases, potentially preserving visual function that might otherwise be lost. Second, it helps in optimizing treatment strategies, ensuring that patients receive appropriately aggressive management based on their individual risk profiles. Finally, accurate progression prediction can improve patient counseling, helping individuals understand their prognosis and the importance of treatment adherence.

Despite advancements in diagnostic technologies and our understanding of glaucoma pathophysiology, predicting disease progression remains challenging. Current methods rely heavily on serial measurements of intraocular pressure (IOP), VF tests, and structural imaging of the optic nerve and retinal nerve fiber layer (RNFL) [5]. However, these approaches have limitations: Glaucoma progression assessment faces several challenges that impact its accuracy and reliability. VF tests, which are crucial in evaluating glaucoma progression, are particularly prone to significant intra- and intertest variability. This variability makes it difficult for clinicians to distinguish between true disease progression and mere test fluctuations, as highlighted by Junoy Montolio et al. [6] in their 2012 study. Furthermore, the interpretation of structural changes observed in imaging studies is inherently subjective, leading to potential inconsistencies in progression assessment among different clinicians. This issue was underscored by Michelessi et al. [7] in their 2015 research.

Another significant challenge lies in the limited integration of known risk factors for glaucoma progression. While various risk factors have been identified, including older age, higher IOP, and thinner central corneal thickness, current prediction models often fail to comprehensively incorporate these factors. This limitation was noted by Saunders, Russell, and Crabb [8] in their 2015 study. Additionally, the multifactorial nature of glaucoma progression, which involves complex interactions between various physiological and environmental factors, presents a formidable challenge. Traditional statistical methods often struggle to capture these complex interactions, further complicating the accurate assessment and prediction of disease progression.

Artificial intelligence (AI), particularly machine learning and deep learning techniques, offers promising solutions to these challenges. AI algorithms can analyze vast amounts of complex, multidimensional data, identifying patterns and relationships that may not be apparent to human observers. In recent years, AI has shown remarkable potential in various aspects of ophthalmology, including the detection of diabetic retinopathy, age-related macular degeneration, and glaucomatous optic neuropathy [9].

In the context of glaucoma progression prediction, AI offers several potential advantages that could significantly enhance the accuracy and effectiveness of assessment methods. AI models have the capability to integrate diverse data types simultaneously, including structural imaging data, functional test results, clinical measurements, and patient demographics. This comprehensive approach allows for a more holistic assessment of progression risk, as demonstrated by Elze et al. [10] in their 2015 study. Furthermore, AI has the potential to reduce subjective variability in glaucoma assessment. By applying consistent criteria across large datasets, AI can mitigate the subjectivity inherent in human interpretation of test results and imaging studies, leading to more standardized and reliable assessments.

Another significant advantage of AI lies in its ability to capture complex interactions between various risk factors. Machine learning algorithms, particularly deep learning models, excel at identifying nonlinear relationships that may not be apparent through traditional statistical methods. This capability can potentially improve the accuracy of glaucoma progression predictions, as highlighted by Li et al. [11] in their 2018 research. Moreover, AI models can be trained on large, diverse patient populations, enabling more personalized risk assessments. These assessments can account for individual patient characteristics and unique disease patterns, providing a level of personalization that is challenging to achieve with conventional methods. This potential for personalized risk assessment was explored by Kazemian et al. [12] in their 2018 study, underscoring the promising role of AI in advancing glaucoma care and management.

This paper aims to present a conceptual framework for an AI-driven personalized glaucoma progression prediction system. We will outline the key components of such a system, discuss its potential clinical applications and impact, and address the challenges and strategies for its implementation in clinical practice. By doing so, we hope to provide a roadmap for future research and development in this critical area of ophthalmology, ultimately contributing to improved patient care and outcomes in glaucoma management.

## 2. Method

### 2.1. Overview of the Framework

The proposed conceptual framework for AI-driven personalized glaucoma progression prediction is designed to leverage the power of AI to enhance clinical decision-making in glaucoma management. This framework aims to address the current limitations in predicting glaucoma progression by integrating diverse data sources, applying advanced machine learning techniques, and providing clinically relevant outputs. The framework consists of four main components: data integration and preprocessing, AI model architecture and training, personalized prediction generation, and a clinical decision support interface.

**Figure 1:**
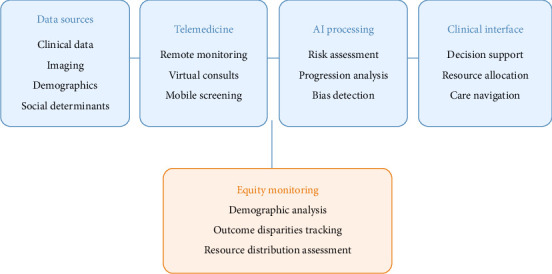
AI-driven glaucoma progression prediction framework with integrated equity monitoring and telemedicine components.

A key consideration in the framework's design is its adaptability to various resource settings and potential integration with telemedicine platforms. This ensures the system can serve diverse populations, from urban centers to remote areas with limited access to specialized care.  Figure 1 aims to reflect these additional considerations. The framework illustrates the interconnected flow of data and processes from initial data collection through clinical implementation, with specific emphasis on equity monitoring and telemedicine integration. The blue components represent the core processing pipeline, while the orange component highlights the continuous equity monitoring system that oversees all processes to ensure fair and equitable outcomes across different populations and resource settings.

### 2.2. Data Integration and Preprocessing

The foundation of the proposed framework is a comprehensive and diverse dataset that captures the multidimensional nature of glaucoma progression while ensuring equitable representation across different populations and healthcare settings. This dataset encompasses structural imaging data (such as optical coherence tomography [OCT] scans of the optic nerve head and RNFL), functional test results (particularly VF tests), clinical measurements (including IOP readings and central corneal thickness), patient demographics, and crucially, social determinants of health data including healthcare access metrics, socioeconomic indicators, and environmental factors that may influence disease progression and treatment adherence. The integration of these diverse data types is crucial for capturing the complex interactions that influence glaucoma progression [13].

Data preprocessing, a critical step in ensuring the quality and consistency of input data, has been designed to accommodate varying levels of resource availability and data quality. The preprocessing pipeline begins with sophisticated data cleaning algorithms that can handle artifacts and outliers while accounting for systematic differences in data quality that may arise from different healthcare settings. This is followed by context-aware normalization procedures that account for differences not only in measurement scales but also in equipment quality and calibration variations commonly found across different clinical settings. For instance, in the case of OCT images, the preprocessing includes robust segmentation algorithms that can handle varying image qualities and thickness measurements [14]. For VF data, preprocessing incorporates reliability indices that account for patient-specific and environment-specific factors that might affect test quality, including the calculation of global indices such as mean deviation and pattern standard deviation, as well as pointwise sensitivity values [15].

To support telemedicine applications and improve accessibility, the preprocessing pipeline includes specialized modules for handling remotely captured data. These modules assess image quality in real time, provide feedback for image capture optimization, and employ adaptive compression techniques to enable efficient data transmission in low-bandwidth settings. The system also supports asynchronous processing, allowing for store-and-forward telemedicine applications in areas with limited connectivity.

A key innovation in the preprocessing stage is the implementation of data harmonization techniques that address the challenge of integrating information from different sources and devices. This harmonization process employs advanced transfer learning techniques and device-independent feature extraction methods to ensure consistent analysis across various platforms and settings. Given the variety of diagnostic equipment used in different clinical settings, it is essential to develop methods for standardizing data across platforms. This might involve the use of transfer learning techniques or the development of device-independent features [16]. The system maintains calibration data for different devices and automatically applies appropriate corrections to ensure comparability of measurements across different clinical settings.

Additionally, the preprocessing pipeline includes mechanisms for identifying and addressing potential sources of bias in the data. This includes monitoring the demographic distribution of the training data, implementing active sampling strategies to ensure representative datasets, and employing specialized algorithms to detect and correct for systematic biases that might arise from differences in healthcare delivery or data collection practices across different populations or settings.

### 2.3. AI Model Architecture and Training

The core of the framework is the AI model designed to predict glaucoma progression. Given the complexity of the task and the diverse nature of the input data, a deep learning approach, specifically a multimodal neural network architecture, is proposed. This architecture would allow for the integration of different data types while capturing complex, nonlinear relationships between various factors influencing glaucoma progression.

The proposed model could consist of separate neural network branches for processing different data types (e.g., convolutional neural networks for imaging data, recurrent neural networks for time-series data like IOP measurements), with these branches converging into fully connected layers for final prediction [17]. This architecture allows the model to learn relevant features from each data type independently while also capturing interactions between different modalities.

Training of the model would require a large, diverse dataset of glaucoma patients with known progression outcomes. The model would be trained to predict future VF loss or structural changes based on baseline and follow-up data. Techniques such as transfer learning could be employed to leverage pretrained models on related tasks, potentially improving performance and reducing the required amount of training data [18].

The model architecture is specifically designed to promote health equity through several key innovations. First, it employs a hierarchical learning approach that can function across different levels of data availability and quality, making it suitable for deployment in diverse healthcare settings. The model can operate with varying levels of input data completeness, gracefully degrading its predictions rather than failing when certain measurements are unavailable—a common scenario in resource-limited settings. This adaptability is achieved through a novel multistream architecture that can process data from high-end diagnostic equipment while also accommodating basic clinical measurements and images from portable devices [19]. To address potential biases, the training process incorporates fairness constraints and demographic parity metrics across different population subgroups. The model employs adversarial debiasing techniques to minimize unwanted correlations between predictions and sensitive demographic attributes while maintaining high predictive accuracy [20]. Regular bias audits during training help identify and mitigate any systematic disparities in model performance across different patient populations.

For resource-limited settings, the framework includes model compression techniques and lightweight architectures that can run on basic hardware while maintaining clinical accuracy. The system employs progressive learning approaches that allow the model to start with basic functionality and gradually expand its capabilities as more data become available. This scalable approach enables healthcare facilities to begin using the system with minimal infrastructure and expand their capabilities over time.

### 2.4. Personalized Prediction Generation

Once trained, the AI model would generate personalized predictions of glaucoma progression for individual patients. These predictions could take various forms, such as the probability of significant VF loss within a specific timeframe, or the expected rate of RNFL thinning. Importantly, the model should provide not just point estimates but also measures of uncertainty, reflecting the confidence level of its predictions [21].

The personalized nature of these predictions is a key feature of the proposed framework. By considering a wide range of individual factors and their complex interactions, the AI model can potentially provide more accurate and tailored predictions than current clinical methods. This personalization could extend to predicting the likely outcomes of different treatment strategies, aiding in treatment selection [12].

The personalization capabilities extend beyond clinical factors to consider social determinants of health and resource availability. The system adjusts its recommendations based on available treatment options, considering factors such as medication costs, access to specialized care, and local healthcare resources. By incorporating these contextual factors, the system can provide more realistic and actionable recommendations across diverse healthcare settings. For instance, in settings where certain advanced diagnostic tools are unavailable, the system can provide alternative assessment strategies while clearly communicating any resulting uncertainties in its predictions.

### 2.5. Clinical Decision Support Interface

The final component of the framework is a clinical decision support interface designed to present the AI-generated predictions in a clear, interpretable format for clinicians. This interface should integrate seamlessly with existing electronic health record systems and clinical workflows. It should provide visual representations of predicted progression, alongside key factors influencing the prediction.

An important feature of this interface would be the ability to explore “what-if” scenarios, allowing clinicians to see how different treatment strategies might affect the predicted progression. This could aid in shared decision-making with patients, helping to illustrate the potential benefits of more aggressive treatment or the risks of nonadherence [22].

The interface should also provide some level of explainability, offering insights into which factors are most influencing the AI's predictions. This explainability is crucial for building trust in the AI system and allowing clinicians to apply their expertise in interpreting and acting on the predictions [23].

This conceptual framework for AI-driven glaucoma progression prediction represents a comprehensive approach to leveraging AI in glaucoma management. By integrating diverse data sources, employing advanced machine learning techniques, and providing clinically relevant, personalized predictions, this framework has the potential to significantly enhance clinical decision-making in glaucoma care. To promote equitable implementation, the interface includes specialized features for different resource settings. In areas with limited specialist access, the interface provides more detailed guidance for general practitioners and primary care providers. The system includes built-in telehealth capabilities for remote specialist consultation when needed. Additionally, the interface incorporates multilingual support and culturally adapted visual elements to enhance accessibility across diverse populations. Regular equity audits analyze system usage patterns and outcomes across different demographic groups and healthcare settings, with results displayed through an equity monitoring dashboard that helps identify and address potential disparities in system performance and clinical outcomes.

## 3. Results

### 3.1. Potential Clinical Applications and Impact

The proposed AI-driven framework for personalized glaucoma progression prediction has the potential to significantly impact clinical practice in ophthalmology. This section explores the various ways in which this advanced predictive tool could enhance patient care, optimize treatment strategies, and contribute to clinical research in glaucoma management.

**Figure 2:**
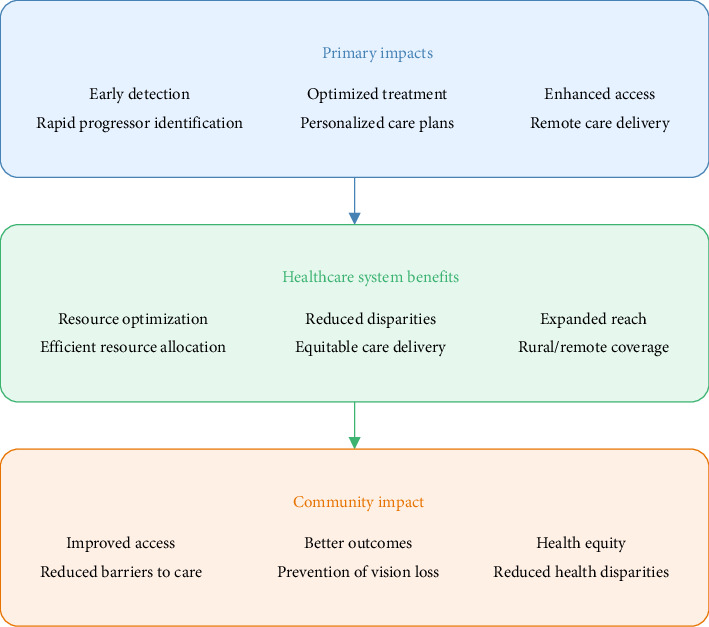
Cascading impact of AI-driven glaucoma progression prediction on health equity.

Figure 2 illustrates the multilevel impact of the AI system, from immediate clinical benefits through systemic healthcare improvements to broader community impact, with a specific focus on advancing health equity across diverse populations and resource settings. The framework's impact on health equity in glaucoma care operates at multiple levels (Figure 2). At the primary level, it enables early detection and intervention across diverse healthcare settings, including resource-limited areas. At the healthcare system level, it optimizes resource allocation and reduces care disparities through standardized, objective assessments. At the community level, it improves access to specialized care and promotes better health outcomes across different demographic groups.

#### 3.1.1. Enhancing Early Detection of Rapid Progressors

One of the most significant potential applications of the AI-driven prediction model is in the early identification of patients at high risk of rapid glaucoma progression, particularly in underserved populations where delayed diagnosis is common. Current clinical methods often struggle to differentiate between slow and fast progressors in the early stages of the disease, leading to potential delays in implementing more aggressive treatment for high-risk patients. This challenge is particularly acute in settings with limited access to specialists or advanced diagnostic equipment.

The AI model addresses these disparities through several mechanisms. First, it can function with varying levels of input data, making it effective even in settings where comprehensive testing may not be available. Second, it incorporates population-specific risk factors and progression patterns, ensuring accurate risk assessment across different demographic groups. Third, it supports task-shifting to nonspecialist healthcare workers through clear risk stratification and decision support, enabling earlier intervention in areas with limited specialist access.

As demonstrated by De Moraes et al. [24], early detection of fast progressors can significantly impact long-term visual outcomes. Their study showed that patients identified as rapid progressors and treated aggressively had a 42% lower risk of VF worsening compared to those identified later. By leveraging AI to enhance this early detection, particularly in underserved populations, clinicians could intervene more promptly with appropriate treatment intensification, potentially preserving visual function that might otherwise be lost. This capability is especially valuable in communities where regular access to eye care specialists is limited.

#### 3.1.2. Optimizing Treatment Strategies

The personalized nature of the AI-driven predictions plays a crucial role in treatment optimization, with particular attention to resource availability and accessibility. By providing predictions of disease progression under different treatment scenarios, the model assists clinicians in selecting the most appropriate and feasible management strategy for each patient's specific context. This capability aligns with both the growing emphasis on personalized medicine in ophthalmology, as highlighted by Weinreb et al. [25], and the need to adapt care strategies to different resource settings.

The system's treatment recommendations are contextually aware, considering not only clinical factors but also practical constraints such as medication costs, availability of different treatment options, and access to follow-up care. For instance, in settings where certain medications may be cost-prohibitive or unavailable, the system suggests alternative evidence-based treatment approaches while clearly communicating their comparative effectiveness.

For determining optimal target IOP, the system accounts for both clinical factors and practical considerations. While general guidelines exist for IOP targets, the ideal target can vary significantly between patients based on various factors. Kazemian et al. [12] demonstrated the potential of machine learning in personalizing IOP targets, showing that individualized targets could lead to better outcomes compared to standard care. The proposed AI framework extends this concept by incorporating socioeconomic factors and healthcare access metrics into its recommendations.

Moreover, the model aids in deciding between different treatment modalities, such as medical therapy versus surgical intervention, while considering the local availability of surgical expertise and follow-up care capabilities. By predicting the likelihood of success for different treatment options based on individual patient characteristics and local healthcare resources, the AI system supports more informed and contextually appropriate decision-making. This could potentially lead to more effective treatment choices, reducing both undertreatment in high-risk patients and overtreatment in low-risk patients, regardless of their geographic or socioeconomic situation.

#### 3.1.3. Improving Patient Counseling and Engagement

The AI-driven prediction model serves as a powerful tool for patient education and engagement across diverse cultural and socioeconomic contexts. Visual representations of predicted disease progression, adapted for varying health literacy levels and cultural backgrounds, along with the ability to demonstrate the potential impact of different treatment options or lifestyle changes, can greatly enhance patient understanding of their condition. This improved understanding is particularly crucial in communities where glaucoma awareness may be limited or where cultural barriers to treatment adherence exist.

The system incorporates culturally sensitive communication strategies and multilingual support to ensure effective patient education across diverse populations. Visual aids and risk communications are designed to be culturally appropriate and accessible to patients with varying levels of health literacy. This comprehensive approach to patient communication addresses a critical gap in glaucoma care, as Sleath et al. [26] found that patients who better understood their glaucoma diagnosis and the importance of treatment were more likely to adhere to their medication regimen.

Furthermore, the ability to show patients the potential long-term consequences of poor adherence in a personalized, culturally sensitive manner serves as a powerful motivational tool. The system also includes features to address common barriers to adherence, such as medication cost concerns, transportation challenges, and cultural beliefs about treatment. This aligns with the findings of Chow et al. [27], who demonstrated that shared decision-making tools in glaucoma care led to improved patient knowledge and satisfaction with care decisions.

To support ongoing engagement, the system provides personalized reminder systems adaptable to patients' preferences and circumstances, including options for mobile phone integration in areas with limited internet connectivity. It can also facilitate connection with community health workers and support groups, recognizing the importance of community-based support in maintaining long-term treatment adherence.

One of the most significant challenges in glaucoma management is ensuring adequate follow-up for individuals identified as at risk, particularly those who lack health insurance, financial resources, or access to specialized care. AI-powered prescreening and risk stratification can enhance early detection, but without a robust care continuity framework, many high-risk patients may remain untreated, exacerbating healthcare disparities. To address this gap, AI-driven glaucoma management should be paired with community-based care models, telemedicine, and public health initiatives to improve follow-up care accessibility. Mobile eye care units and community health workers can play a pivotal role in reaching patients in underserved areas, conducting regular screenings, monitoring disease progression, and facilitating referrals. In settings where in-person ophthalmologic care is limited, teleophthalmology platforms can connect high-risk patients with specialists for remote consultations, reducing the need for frequent travel.

Cost barriers remain a significant obstacle for uninsured or underinsured patients. Government-subsidized programs, nonprofit healthcare initiatives, and hospital-supported financial assistance programs can help provide affordable or free follow-up care for glaucoma suspects. AI-driven triage systems can also assist in prioritizing high-risk patients for subsidized care, ensuring that those with the greatest need receive timely intervention. Additionally, AI-powered decision support tools can be integrated into primary care settings, allowing nonspecialist providers to monitor and manage stable glaucoma suspects. By equipping general practitioners and optometrists with AI-assisted diagnostics, follow-up care can be provided outside traditional ophthalmology clinics, expanding access for those who may otherwise be lost to follow-up.

Future research should focus on developing sustainable care models that integrate AI screening with long-term patient monitoring, particularly for economically disadvantaged and geographically isolated populations. AI must not only enhance early detection but also facilitate continuous, accessible, and affordable care pathways, ensuring that diagnosed patients receive the follow-up they need to prevent irreversible vision loss.

#### 3.1.4. Supporting Clinical Research and Drug Development

Beyond its direct clinical applications, the AI-driven prediction framework has significant implications for advancing health equity through research and drug development. By analyzing large datasets across diverse populations, the AI system helps identify previously underrecognized progression patterns and risk factors specific to different demographic groups, potentially leading to more inclusive and representative glaucoma research.

In the context of clinical trials, the AI system plays a valuable role in ensuring diverse patient representation and equitable access to research opportunities. By accurately identifying patients at high risk of progression across different populations, it helps in enrolling more representative participant groups for trials of new therapies, addressing a historical bias in clinical research toward certain demographic groups. This application aligns with the concept of “prognostic enrichment” in clinical trials, as described by Temple [28], while promoting more inclusive research practices.

The framework also supports the development of population-specific therapeutic approaches by identifying differential treatment responses across various demographic groups. This capability is particularly valuable for developing targeted interventions for populations that have historically been underrepresented in glaucoma research. The system's ability to analyze treatment outcomes across diverse populations can help identify potential genetic or environmental factors that influence treatment efficacy, leading to more personalized and effective therapeutic strategies.

Additionally, the framework facilitates research into healthcare delivery disparities by tracking outcomes across different healthcare settings and populations. This includes analyzing the impact of social determinants of health on glaucoma progression and treatment outcomes, helping to identify systemic barriers to care that may need to be addressed through policy or programmatic interventions. The system's capacity to aggregate and analyze real-world data from diverse clinical settings provides valuable insights for health services research and policy development aimed at reducing healthcare disparities.

### 3.2. Implementation Challenges and Strategies

The implementation of an AI-driven glaucoma progression prediction system in clinical practice presents several challenges that must be addressed, particularly when considering the goal of promoting health equity across diverse healthcare settings. This section discusses these challenges and proposes strategies to overcome them, ensuring successful integration and adoption of this technology in ophthalmology while minimizing the risk of exacerbating existing healthcare disparities.

#### 3.2.1. Data Quality and Standardization

One of the primary challenges in implementing AI-driven systems in health care is ensuring the quality and standardization of data while maintaining representativeness across diverse populations and healthcare settings. In the context of glaucoma progression prediction, this challenge is particularly pronounced due to the varying quality of diagnostic equipment, different levels of healthcare provider expertise, and disparate documentation practices across different clinical settings.

Data quality issues can arise from various sources, including measurement errors, inconsistent recording practices, and missing data, with these challenges often being more pronounced in resource-limited settings. These issues can significantly impact the performance and reliability of AI models and potentially lead to biased predictions that could worsen healthcare disparities. As noted by Ting et al. [9], the quality of input data is crucial for the development of robust AI algorithms in ophthalmology.

To address the challenge of data quality and consistency while promoting equity, several strategies have been developed. First, standardized data collection protocols have been designed with flexibility to accommodate different resource levels. These protocols include guidelines for performing diagnostic tests with both advanced and basic equipment, ensuring that facilities with limited resources can still contribute valuable data to the system. The protocols also include specific guidance for documenting social determinants of health and healthcare access factors that may impact glaucoma care.

The implementation of automated data quality assessment tools has been adapted to different clinical contexts. These tools employ context-aware validation rules that consider local resources and practices while maintaining essential quality standards. Machine learning techniques, as demonstrated by Wang et al. [29], are leveraged to develop sophisticated data cleaning algorithms that learn from patterns in high-quality data to correct or impute problematic data points, with special consideration for systematic differences that may exist across different healthcare settings.

Cross-platform data harmonization is crucial given the variety of diagnostic equipment used in ophthalmology. The framework includes specialized algorithms for harmonizing data across different platforms and equipment types, ensuring that measurements from basic diagnostic tools can be meaningfully compared with those from advanced equipment. This approach, building on the work of Medeiros, Jammal, and Thompson [30], enables the creation of more inclusive and representative datasets while maintaining clinical validity.

Collaborative data sharing initiatives are structured to promote equity in data representation. These initiatives include targeted data collection efforts in underserved communities and incentive structures that encourage participation from diverse healthcare settings. However, these efforts are carefully balanced with patient privacy concerns and regulatory requirements, with special attention to protecting vulnerable populations. The framework draws inspiration from successful collaborative initiatives like the OASIS project in neuroimaging, as described by Marcus et al. [31], while incorporating additional protections and considerations for health equity.

A crucial aspect of AI-driven glaucoma management is the quality and accessibility of the instruments used to acquire data. Current VF tests and retinal imaging devices, while highly accurate, are often expensive and bulky, and require specialized personnel for operation. This limits their availability in resource-constrained settings, contributing to disparities in glaucoma diagnosis and management.

To ensure widespread and equitable adoption of AI solutions, there is a pressing need for affordable, portable, and durable diagnostic tools that offer comparable accuracy to high-end clinical devices. Advances in low-cost, smartphone-based retinal imaging, tablet-based perimetry, and portable OCT devices are promising solutions. Studies have demonstrated that portable fundus cameras and AI-assisted VF tests can provide reliable assessments, making them viable alternatives in primary care and rural settings [30].

AI systems must also be designed to function with heterogeneous data sources, ensuring that predictions remain robust even when diagnostic quality varies. This requires standardization algorithms that calibrate data from different devices to maintain comparability, adaptive AI models that adjust confidence levels based on data quality from different instruments, and validation of AI performance using diverse imaging sources, including lower-cost and portable diagnostic tools. The framework proposed in this paper is designed to integrate multiple levels of data fidelity, ensuring usability across diverse healthcare settings. Future research should explore how low-cost imaging and perimetry solutions can be optimized for AI-based glaucoma assessment, making advanced diagnostics accessible to underserved populations without compromising reliability.

The framework proposed in this paper is designed to integrate multiple levels of data fidelity, ensuring usability across diverse healthcare settings. Future research should explore how low-cost imaging and perimetry solutions can be optimized for AI-based glaucoma assessment, making advanced diagnostics accessible to underserved populations without compromising reliability.

#### 3.2.2. Model Interpretability and Clinical Trust

The “black box” nature of many advanced AI models poses a significant challenge to their adoption in clinical practice, particularly in settings where healthcare providers may have limited experience with AI technologies. This challenge becomes even more critical when considering the need to build trust across diverse cultural contexts and healthcare settings.

Addressing the challenge of interpretability in AI-driven glaucoma progression prediction requires a culturally sensitive and context-aware approach. One fundamental strategy involves the development of interpretable AI techniques that can explain their decisions in ways that are meaningful to both healthcare providers and patients from diverse backgrounds. While deep learning models are powerful, they must be complemented by more transparent approaches, especially for aspects of the prediction task where the underlying relationships are relatively simple. This approach aligns with [32] study, emphasizing the importance of model transparency in high-stakes decision-making contexts like health care.

For complex models where simpler techniques may not suffice, the system employs culturally adapted post hoc explanation methods. Techniques such as SHAP (Shapley additive explanations) values or LIME (local interpretable model-agnostic explanations) are implemented with modifications to accommodate different levels of AI literacy among healthcare providers. These methods not only explain which factors influence the model's predictions but also contextualize these explanations within local healthcare practices and resources.

Visualization tools play a crucial role in enhancing interpretability across different cultural and educational backgrounds. The system includes customizable visualization interfaces that can be adapted to different cultural contexts and levels of technical expertise. These visualizations help clinicians understand the model's decision-making process while respecting local medical practices and cultural norms.

#### 3.2.3. Integration With Existing Clinical Workflows

The successful integration of AI-driven glaucoma progression prediction into existing clinical workflows requires careful consideration of diverse healthcare settings, from well-resourced urban centers to remote rural clinics. This integration presents several challenges, including variations in clinical resources, staffing patterns, and technological infrastructure.

To integrate AI systems into clinical workflows effectively while promoting health equity, several strategies have been developed. User-centered design principles are applied with specific attention to different resource settings and cultural contexts. This approach, as highlighted by Cai et al. [33], involves extensive consultation with healthcare providers from diverse practice settings to ensure the system meets their needs and respects local care delivery patterns.

The implementation of a modular system architecture allows for flexible deployment across different resource levels. This approach enables healthcare facilities to start with basic functionality and gradually expand their capabilities as resources permit. For facilities with limited internet connectivity, the system includes offline functionality and asynchronous data synchronization capabilities.

Comprehensive training programs are designed to accommodate different levels of technological literacy and clinical expertise. These programs include both technical training on system use and clinical guidance on incorporating AI-driven insights into patient care decisions. Training materials are culturally adapted and available in multiple languages to ensure accessibility across diverse healthcare settings.

AI-powered prescreening and automated risk stratification have the potential to increase patient volumes in healthcare settings by identifying at-risk individuals who might otherwise remain undiagnosed. While this early detection is critical for preventing vision loss, it raises concerns about whether current healthcare systems can accommodate a potential surge in patient demand without compromising quality of care.

To manage this challenge, AI-driven glaucoma care must be integrated strategically into healthcare workflows to optimize resource allocation and avoid overwhelming specialists. Key strategies may include the following:

Task-shifting to nonspecialist providers—AI-assisted triage systems can help primary care physicians, optometrists, and trained technicians conduct initial risk assessments, referring only high-risk cases to ophthalmologists. This reduces specialist burden while expanding access to care in underserved areas.

Telemedicine integration—AI-driven prescreening can be paired with teleophthalmology to increase capacity without increasing physical patient loads. Remote AI-assisted evaluations can prioritize urgent cases while allowing stable or low-risk patients to be monitored virtually.

AI-augmented decision support—AI models can provide automated reports and risk stratifications, allowing clinicians to quickly review AI-processed data instead of manually analyzing extensive patient records. This improves efficiency and reduces administrative burden.

Dynamic scheduling and workflow optimization—AI-driven prescreening can be combined with predictive analytics to anticipate patient inflow trends, enabling better workforce planning and appointment scheduling.

Hybrid models of care—Healthcare systems should gradually phase in AI prescreening, allowing clinics to adjust resources while monitoring its impact on clinician workload and patient outcomes.

By implementing task-shifting, telemedicine, and workflow optimization, healthcare systems can harness the benefits of AI-driven prescreening without sacrificing care quality. Future research should evaluate longitudinal impacts on clinician workload and explore scalable AI deployment models for different healthcare settings.

#### 3.2.4. Regulatory Considerations and Approval Processes

The regulatory landscape for AI in health care presents unique challenges when considering deployment across different healthcare systems and jurisdictions. Navigating these regulatory processes while ensuring equitable access to the technology requires a nuanced approach that considers varying regulatory requirements and healthcare system capacities.

Early and ongoing engagement with regulatory bodies in different jurisdictions helps ensure compliance while advocating for frameworks that promote equitable access. The system's development follows the FDA's Digital Health Software Precertification (Pre-Cert) Program principles while incorporating flexibility to meet diverse regulatory requirements in different regions.

Documentation and testing procedures are designed to demonstrate safety and efficacy across diverse patient populations and healthcare settings. This includes comprehensive testing in different resource contexts and across various demographic groups to ensure equitable performance. Postmarket surveillance systems are implemented with specific attention to monitoring outcomes across different populations and healthcare settings.

Obtaining FDA clearance and regulatory approval for AI-driven medical technologies is indeed a costly and time-consuming process, as it involves extensive validation, clinical trials, and compliance with evolving regulatory frameworks. These costs arise from software development, data collection, third-party audits, and ongoing postmarket surveillance. A key question in the adoption of AI-driven glaucoma management systems is who bears the financial burden of regulatory approval? Typically, the costs are distributed among multiple stakeholders. Private sector technology developers and AI companies often fund initial research and regulatory submissions, especially for commercially driven AI applications. Academic institutions and research consortia may secure public or philanthropic grants to develop noncommercial AI models. Healthcare providers and insurance systems may also play a role, as regulatory-compliant AI solutions can ultimately reduce long-term costs by improving patient outcomes and reducing preventable blindness. Additionally, government agencies and nonprofit organizations may offer funding incentives for AI solutions that improve healthcare accessibility, particularly in underserved regions.

In the case of AI-driven glaucoma prediction models, a sustainable cost-sharing strategy is essential. One approach is public–private partnerships (PPPs), where academic research institutions collaborate with commercial AI firms and regulatory bodies to cofund compliance efforts while maintaining ethical oversight. Another avenue is value-based reimbursement models, where AI technologies demonstrating clear clinical benefits and cost savings could qualify for reimbursement by insurance providers, thus incentivizing investment in regulatory approval. Ultimately, ensuring that AI solutions are both financially sustainable and accessible requires multistakeholder engagement. This includes exploring alternative funding models such as regulatory sandboxes, where AI solutions can be tested in controlled clinical environments before full-scale approval, reducing financial barriers to market entry.

Beyond instrument standardization, ensuring that AI models perform reliably across diverse populations requires rigorous validation using local datasets. This process is critical to identifying potential biases and mitigating risks that could disproportionately affect certain demographic groups. However, testing AI models on region-specific patient data involves substantial costs related to data collection, annotation, computational infrastructure, and clinical validation.

The financial responsibility for these validation efforts is often distributed among multiple stakeholders. AI developers and technology companies typically fund local testing as part of regulatory compliance requirements, ensuring that models meet performance benchmarks across different populations. Public healthcare institutions and government agencies may also provide financial support, particularly when AI systems are integrated into national health programs aimed at improving equity in disease detection and management. International organizations and research funding bodies, such as the World Health Organization and the National Institutes of Health, frequently invest in AI validation studies for underserved regions to promote global healthcare equity. Large hospital networks and medical consortia may contribute funding, especially when AI models are deployed within their clinical settings.

PPPs present an effective cost-sharing approach, allowing collaboration between AI companies, healthcare providers, and regulatory bodies to ensure rigorous validation while distributing financial burdens. Additionally, privacy-preserving approaches such as federated learning reduce the need for extensive data centralization, enabling AI training on decentralized datasets without violating privacy regulations. These strategies help minimize validation costs while maintaining transparency, fairness, and high clinical safety standards in AI-driven glaucoma management.

#### 3.2.5. Ethical Considerations and Patient Privacy

Ethical implementation of AI in healthcare requires careful attention to equity, fairness, and cultural sensitivity while maintaining robust privacy protections. The framework addresses these considerations through several key strategies.

Comprehensive data governance policies are established with specific attention to protecting vulnerable populations and respecting cultural differences in privacy expectations. These policies ensure compliance with various international privacy regulations while incorporating additional protections for underserved communities. The framework also includes robust bias detection and mitigation processes that regularly assess system performance across different demographic groups and healthcare settings. As highlighted by Rajkomar et al. [34], this includes monitoring for potential disparities in system performance and implementing corrective measures when needed.

Beyond fairness and privacy, another critical concern is the safety of AI-driven clinical decision-making. The reliance on deep learning models, while promising, introduces challenges such as automation bias—where clinicians may over-rely on AI-generated predictions without critically assessing their validity. To mitigate this risk, the proposed framework ensures that AI remains an assistive tool rather than a replacement for human judgment. AI-generated recommendations are presented with confidence scores and explainability features that help clinicians interpret the model's outputs within the broader clinical context.

The interpretability of AI models is essential to fostering clinician trust and ensuring safe integration into practice. Many deep learning models function as “black boxes,” making it difficult for users to understand how specific predictions are made. To address this, the framework incorporates explainable AI (XAI) techniques, such as SHAP and LIME, which highlight the most influential factors driving AI-generated predictions. These explainability tools allow clinicians to validate predictions against their clinical expertise, reducing the risk of blind trust in AI outputs.

Community engagement plays a crucial role in ensuring ethical implementation. The framework includes structured processes for obtaining input from diverse stakeholders, including patient advocates, community leaders, and healthcare providers from various practice settings. This helps ensure that the system's deployment respects local values and addresses community needs while promoting health equity.

Furthermore, AI adoption in clinical settings must comply with safety regulations and industry standards. Existing regulatory frameworks, such as the FDA's Digital Health Software Precertification Program and the EU Medical Device Regulation (MDR), provide guidance for evaluating the safety and efficacy of AI-driven healthcare technologies. The proposed system adheres to these regulatory requirements by incorporating ongoing model validation, bias audits, and postmarket performance monitoring to ensure its safety and effectiveness across diverse populations.

Regular ethical reviews assess the system's impact on healthcare disparities and identify opportunities for improving equitable access. These reviews consider not only technical performance but also broader social impacts, including effects on healthcare access and outcomes across different populations.

Moreover, the implementation of AI in health care requires substantial investment in cybersecurity and data protection infrastructure to comply with regulatory requirements (e.g., GDPR, HIPAA) and safeguard sensitive patient information. Protecting healthcare data from cyber threats, unauthorized access, and potential breaches demands continuous updates to security protocols, encrypted data storage, and secure transmission frameworks, all of which introduce significant financial considerations.

The responsibility for funding cybersecurity and infrastructure investments is typically shared across multiple stakeholders.

1. Healthcare institutions and providers—Large hospitals and healthcare systems often allocate budgets for cybersecurity as part of their broader IT investments. However, smaller clinics and resource-limited settings may lack sufficient funding, creating a risk of unequal data protection standards.
2. AI developers and technology vendors—Companies offering AI-driven healthcare solutions must integrate robust security features into their systems. Some AI firms cover security costs as part of software licensing agreements, ensuring compliance with industry security standards.
3. Government and regulatory bodies—Many governments provide funding and incentives to support cybersecurity in health care, particularly in public health initiatives. Investment in national-level secure cloud solutions for medical AI applications can reduce costs for individual providers.
4. PPPs—Collaborations between governments, nonprofits, and private technology firms can create shared cybersecurity infrastructures that provide affordable and scalable data protection solutions.

A sustainable cost-sharing model is essential to prevent cybersecurity investment from becoming a barrier to AI adoption. Innovative solutions, such as federated learning, which processes AI models without centralizing patient data, can reduce cybersecurity risks while minimizing infrastructure costs. Future policies should explore subsidized security frameworks for smaller healthcare facilities, ensuring equitable access to AI-driven healthcare innovations.

## 4. Conclusion

### 4.1. Summary of the Proposed Framework and Its Potential Impact

The AI-driven framework for personalized glaucoma progression prediction presented in this paper represents a significant advancement toward achieving health equity in glaucoma care. By leveraging AI to integrate and analyze diverse data sources while explicitly considering accessibility and equity, this framework has the potential to democratize access to high-quality glaucoma care across different healthcare settings and populations.

The proposed system addresses many of the limitations of current prediction methods while specifically targeting healthcare disparities. By incorporating a wide range of factors that influence glaucoma progression, including social determinants of health and healthcare access metrics, the AI model provides more accurate and contextually appropriate predictions than traditional methods. The framework's ability to function across different resource levels, from basic clinical measurements to advanced imaging data, makes it particularly valuable for reducing disparities in care delivery.

The potential impact of this framework on clinical practice and health equity is substantial. In well-resourced settings, it enhances the precision of care delivery and optimizes resource utilization. In resource-limited settings, it enables evidence-based care delivery even when specialist expertise is not readily available. The system's ability to support task-shifting to nonspecialist healthcare workers, coupled with its telemedicine capabilities, helps extend specialized glaucoma care to underserved populations.

Furthermore, the framework's potential to support clinical research and drug development could accelerate the development of more equitable treatment approaches. By ensuring diverse representation in research data and identifying population-specific treatment responses, it contributes to the development of more inclusive and effective therapeutic strategies.

### 4.2. Future Research Directions

While the proposed framework represents a significant advance in promoting health equity in glaucoma care, several areas warrant further research and development:

The incorporation of genetic data into the prediction model remains a crucial area for future research. Recent studies have identified several genetic variants associated with glaucoma risk and progression [35]. Integrating genetic information with clinical and imaging data could help explain and address disparities in disease progression across different populations, leading to more targeted interventions for specific demographic groups.

Advanced imaging modalities, such as optical coherence tomography angiography (OCTA), present opportunities for enhancing the system's diagnostic capabilities. However, research is needed to develop approaches that maintain diagnostic accuracy when such advanced imaging is not available. As highlighted by Bekkers et al. [36], OCTA provides detailed information about retinal and optic nerve head vasculature, but methods must be developed to derive similar insights from more widely available imaging modalities.

The application of federated learning techniques offers promising solutions for building more representative AI models while protecting patient privacy. This approach allows for the training of AI models across multiple institutions without centralizing patient data, as described by Rieke et al. [37]. Future research should focus on developing federated learning protocols that can accommodate varying data quality and availability across different healthcare settings while maintaining model performance.

Studies on implementation strategies across different resource settings are crucial. This includes research on effective training approaches for healthcare providers with varying levels of technological literacy, strategies for sustainable technology deployment in resource-limited settings, and methods for measuring and optimizing the system's impact on health equity.

Long-term studies will be essential to validate the framework's impact on reducing healthcare disparities. These studies should assess not only the accuracy of progression predictions but also their effect on clinical outcomes and healthcare access across different demographic groups and resource settings.

Looking to the future, we envision this AI framework as part of a comprehensive approach to achieving health equity in glaucoma care. The system could evolve to incorporate real-time monitoring of healthcare disparities, automated adjustment of care recommendations based on local resources and constraints, and direct support for policy interventions aimed at reducing disparities in eye care access and outcomes.

Future developments might include integration with community health worker programs, support for population health management in underserved communities, and enhanced capabilities for identifying and addressing systemic barriers to care. The framework could also expand to support training and capacity building in resource-limited settings, helping to address the global shortage of eye care specialists.

However, as we move toward this AI-enabled future, it remains crucial to maintain a human-centered, equity-focused approach to care. The role of AI should be to augment and support clinical decision-making while helping to reduce, rather than exacerbate, existing healthcare disparities. The empathy, cultural sensitivity, and contextual understanding that human healthcare providers bring to patient care will remain irreplaceable.

In conclusion, the AI-driven framework for glaucoma progression prediction presented in this paper represents an important step toward achieving health equity in eye care. While challenges remain, the potential benefits in terms of improved access to care, better health outcomes, and reduced healthcare disparities are substantial. As we continue to refine and expand these AI systems, we move closer to a future where high-quality glaucoma care is accessible to all, regardless of geographic location, socioeconomic status, or healthcare setting.”

## Data Availability

No data are generated or used in the paper.

## References

[1] Tham Y. C., Li X., Wong T. Y., Quigley H. A., Aung T., Cheng C. Y. (2014). Global Prevalence of Glaucoma and Projections of Glaucoma Burden Through 2040: A Systematic Review and Meta-Analysis. *Ophthalmology*.

[2] Weinreb R. N., Aung T., Medeiros F. A. (2014). The Pathophysiology and Treatment of Glaucoma: A Review. *JAMA*.

[3] Tielsch J. M., Sommer A., Katz J., Royall R. M., Quigley H. A., Javitt J. (1991). Racial Variations in the Prevalence of Primary Open-Angle Glaucoma: The Baltimore Eye Survey. *JAMA*.

[4] Zhang X., Cotch M. F., Ryskulova A. (2012). Vision Health Disparities in the United States by Race/Ethnicity, Education, and Economic Status: Findings From Two Nationally Representative Surveys. *American Journal of Ophthalmology*.

[5] Clement C., Goldberg I., Hatanaka M., Hatanaka M. (2017). Applications of the Water Drinking Test in Glaucoma Management. *Clinical and Experimental Ophthalmology*.

[6] Junoy Montolio F. G., Wesselink C., Gordijn M., Jansonius N. M. (2012). Factors That Influence Standard Automated Perimetry Test Results in Glaucoma: Test Reliability, Technician Experience, Time of Day, and Season. *Investigative Opthalmology & Visual Science*.

[7] Michelessi M., Lucenteforte E., Oddone F. (2015). Optic Nerve Head and Fibre Layer Imaging for Diagnosing Glaucoma. *Cochrane Database of Systematic Reviews*.

[8] Saunders L. J., Russell R. A., Crabb D. P. (2015). Measurement Precision in a Series of Visual Fields Acquired by the Standard and Fast Versions of the Swedish Interactive Thresholding Algorithm: Analysis of Large-Scale Data From Clinics. *JAMA ophthalmology*.

[9] Ting D. S. W., Peng L., Varadarajan A. V. (2019). Deep Learning in Ophthalmology: The Technical and Clinical Considerations. *Progress in Retinal and Eye Research*.

[10] Elze T., Pasquale L. R., Shen L. Q., Chen T. C., Wiggs J. L., Bex P. J. (2015). Patterns of Functional Vision Loss in Glaucoma Determined With Archetypal Analysis. *Journal of the Royal Society Interface*.

[11] Li F., Wang Z., Qu G. (2018). Automatic Differentiation of Glaucoma Visual Field From Non-Glaucoma Visual Filed Using Deep Convolutional Neural Network. *BMC Medical Imaging*.

[12] Kazemian P., Lavieri M. S., Van Oyen M. P., Andrews C., Stein J. D. (2018). Personalized Prediction of Glaucoma Progression Under Different Target Intraocular Pressure Levels Using Filtered Forecasting Methods. *Ophthalmology*.

[13] Medeiros F. A., Zangwill L. M., Anderson D. R. (2012). Estimating the Rate of Retinal Ganglion Cell Loss in Glaucoma. *American Journal of Ophthalmology*.

[14] Xu Y., Yan K., Kim J. (2017). Dual-Stage Deep Learning Framework for Pigment Epithelium Detachment Segmentation in Polypoidal Choroidal Vasculopathy. *Biomedical Optics Express*.

[15] Wu Z., Saunders L. J., Zangwill L. M., Daga F. B., Crowston J. G., Medeiros F. A. (2017). Impact of Normal Aging and Progression Definitions on the Specificity of Detecting Retinal Nerve Fiber Layer Thinning. *American Journal of Ophthalmology*.

[16] Dixit A., Yohannan J., Boland M. V. (2021). Assessing Glaucoma Progression Using Machine Learning Trained on Longitudinal Visual Field and Clinical Data. *Ophthalmology*.

[17] Ardila D., Kiraly A. P., Bharadwaj S. (2019). End-to-End Lung Cancer Screening With Three-Dimensional Deep Learning on Low-Dose Chest Computed Tomography. *Nature Medicine*.

[18] Russakovsky O., Deng J., Su H. (2015). Imagenet Large Scale Visual Recognition Challenge. *International Journal of Computer Vision*.

[19] Gulshan V., Peng L., Coram M. (2016). Development and Validation of a Deep Learning Algorithm for Detection of Diabetic Retinopathy in Retinal Fundus Photographs. *Jama*.

[20] Zhang B. H., Lemoine B., Mitchell M. Mitigating Unwanted Biases With Adversarial Learning.

[21] Abdar M., Pourpanah F., Hussain S. (2021). A Review of Uncertainty Quantification in Deep Learning: Techniques, Applications and Challenges. *Information Fusion*.

[22] Tseng V. L., Yu F., Lum F., Coleman A. L. (2012). Risk of Fractures Following Cataract Surgery in Medicare Beneficiaries. *JAMA*.

[23] Tjoa E., Guan C. (2021). A Survey on Explainable Artificial Intelligence (Xai): Toward Medical Xai. *IEEE Transactions on Neural Networks and Learning Systems*.

[24] De Moraes C. G., Liebmann J. M., Liebmann C. A., Susanna Jr R., Tello C., Ritch R. (2018). Visual Field Progression Outcomes in Glaucoma Subtypes. *Acta Ophthalmologica*.

[25] Weinreb R. N., Leung C. K., Crowston J. G. (2020). Primary Open-Angle Glaucoma. *Nature Reviews Disease Primers*.

[26] Sleath B., Blalock S., Covert D. (2011). The Relationship Between Glaucoma Medication Adherence, Eye Drop Technique, and Visual Field Defect Severity. *Ophthalmology*.

[27] Chow J. T., Hutnik C. M., Solo K., Malvankar-Mehta M. S., Chen J. (2016). When Is Evidence Enough Evidence? A Systematic Review and Meta-Analysis of the Trabectome as a Solo Procedure in Patients With Primary Open-Angle Glaucoma. *Journal of Glaucoma*.

[28] Temple R. (2010). Enrichment of Clinical Study Populations. *Clinical Pharmacology & Therapeutics*.

[29] Wang T., Ke H., Zheng X., Wang K., Sangaiah A. K., Liu A. (2020). Big Data Cleaning Based on Mobile Edge Computing in Industrial Sensor-Cloud. *IEEE Transactions on Industrial Informatics*.

[30] Medeiros F. A., Jammal A. A., Thompson A. C. (2019). From Machine to Machine: An OCT-Trained Deep Learning Algorithm for Objective Quantification of Glaucomatous Damage in Fundus Photographs. *Ophthalmology*.

[31] Marcus D. S., Wang T. H., Parker J., Csernansky J. G., Morris J. C., Buckner R. L. (2007). Open Access Series of Imaging Studies (OASIS): Cross-Sectional MRI Data in Young, Middle Aged, Nondemented, and Demented Older Adults. *Journal of Cognitive Neuroscience*.

[32] Rudin C. (2019). Stop Explaining Black Box Machine Learning Models for High Stakes Decisions and Use Interpretable Models Instead. *Nature Machine Intelligence*.

[33] Cai C. J., Winter S., Steiner D., Wilcox L., Terry M. (2019). ‘Hello AI’: Uncovering the Onboarding Needs of Medical Practitioners for Human-AI Collaborative Decision-Making. *Proceedings of the ACM on Human-Computer Interaction*.

[34] Rajkomar A., Hardt M., Howell M. D., Corrado G., Chin M. H. (2018). Ensuring Fairness in Machine Learning to Advance Health Equity. *Annals of Internal Medicine*.

[35] Gharahkhani P., Jorgenson E., Hysi P. (2021). Genome-Wide Meta-Analysis Identifies 127 Open-Angle Glaucoma Loci With Consistent Effect Across Ancestries. *Nature Communications*.

[36] Bekkers A., Borren N., Ederveen V. (2020). Microvascular Damage Assessed by Optical Coherence Tomography Angiography for Glaucoma Diagnosis: A Systematic Review of the Most Discriminative Regions. *Acta Ophthalmologica*.

[37] Rieke N., Hancox J., Li W. (2020). The Future of Digital Health With Federated Learning. *NPJ Digital Medicine*.

